# Health Related Quality of Life (HRQoL) after transcatheter aortic valve implantation in aortic stenosis patients: exploring a novel threshold for clinically significant improvement after 12 months

**DOI:** 10.1186/s41687-025-00894-1

**Published:** 2025-05-26

**Authors:** Marte Sævik, Marit Helen Andersen, Jan Otto Beitnes, Lars Aaberge, Per Steinar Halvorsen

**Affiliations:** 1 The Intervention Centre, Rikshospitalet, Oslo University Hospital, Oslo, Norway; 2 Faculty of Medicine, University of Oslo, Oslo, Norway; 3 Department of Medicine, Lovisenberg Diaconal Hospital, Oslo, Norway; 4 Department for Interdisciplinary Health Sciences, University of Oslo, Oslo, Norway; 5 Department of Cardiology, Oslo University Hospital, Rikshospitalet, Oslo, Norway

**Keywords:** Aortic stenosis (AS), Transcatheter aortic valve implantation (TAVI), Health related quality of life (HRQoL), SF-36, Minimal clinically important difference (MCID)

## Abstract

**Aim:**

The study aimed to determine the proportion of patients with significant improvements in Health-Related Quality of Life (HRQoL) 12 months after Transcatheter Aortic Valve Implantation (TAVI), using a threshold for physical function (PF) and physical role (RP) domains of the SF-36 questionnaire. Additionally, we explored shared baseline characteristics of patients reporting these improvements.

**Methodology:**

In this prospective observational study, 88 patients with symptomatic, severe aortic stenosis (AS) and preserved ejection fraction were enrolled between April 2017 and February 2020. Exclusion criteria were clinical instability, pacemaker, chronic AF, comorbidities with life expectancy < 1 year. HRQoL was evaluated before and 12 months after transfemoral TAVI using the Norwegian version 2.0 of SF-36, and presented as mean (95% confidence interval). Other outcome measures were 6-minute walking test and NYHA- classification. Independent samples t-test or Mann-Whitney U test was used for between-group comparisons as appropriate. Logistic regression or Chi2-test were used to explore associations between changes in PF and RP and clinical parameters. Statistical significance was set at *p* ≤ 0.05, and clinically significant changes in HRQoL were defined as increase of ≥ 15 points in the PF and RP categories.

**Results:**

Mean age of the cohort was 80 ± 6 years. 44 (50%) patients reported clinically significant improvement in PF, and 46 (52%) in the RP domain. Baseline scores were significantly lower in patients reporting clinical improvement after TAVI, with PF scores pre intervention 43.07 (37.37–48.78) vs. 65.34 (59.01–71.68), *p* < 0.001, and RP 36.01 (29.56–42.46) vs. 59.92 (50.91–68.92), *p* < 0.001. No significant associations were found between improvement in domain scores and parameters from the routine baseline examination, but having ≥ 15-point improvement correlated to baseline PF and RP scores.

**Conclusion:**

Our study highlights the importance of defining a uniform threshold for clinically significant improvement in the SF-36 HRQoL questionnaire for patients undergoing TAVI. Half of the patients reported favorable long-term outcome for PF and RP aspects of SF-36. This emphasizes the importance of HRQoL assessment in the preoperative work up for patients undergoing TAVI.

**Trial registration:**

https://www.clinicaltrials.gov/ 05.04.2017 with ID NCT03107923.

**Supplementary Information:**

The online version contains supplementary material available at 10.1186/s41687-025-00894-1.

## Abstract (plain language summary)

Severe aortic stenosis is in the elderly treated with transcatheter aortic valve implantation, aiming to improve survival and relieve symptoms. Health- related quality of life is an important outcome to measure after the procedure. However, there are no defined values for what constitutes a significant improvement in this patient group when measuring quality of life using the widely used health-related quality of life questionnaire SF-36.

Our study aimed to investigate how many patients had clinically important improvement in physical aspects of quality of life one year after valve implantation. Eighty-eight patients with an average age of 80 years were included. We proposed a new threshold of 15 points for improvement in the physical function and physical role domains of the questionnaire. We found that half of the patients improved in quality of life, and those who improved had lower scores before surgery.

The study emphasizes the need for a clear standard for improvement in quality of life in these patients, highlighting the value of assessment of quality of life before and after TAVI.

**Supplementary Information:**

The online version contains supplementary material available at 10.1186/s41687-025-00894-1.

## Background

Patients diagnosed with severe aortic stenosis (AS) often experience a reduced health-related quality of life (HRQoL) due to the disease itself, accompanying comorbidities, and advanced age [1–3]. Transaortic valve implantation (TAVI) is the recommended treatment for this condition in elderly > 75 years as it compared to surgical aortic valve replacement (SAVR) is less invasive and is associated with lower procedural risks, while offering comparable improvements in survival, symptom relief, and health-related quality of life (HRQoL) [4–7].

Despite the significance of assessing HRQoL in AS patients, there still is no standardized assessment. There are many different questionnaires in use, including the Short-Form 36 Health Survey (SF-36), the Toronto Aortic Stenosis QoL Questionnaire (TASQ), and the Kansas City Cardiomyopathy Questionnaire (KCCQ), with notable diversity in reported endpoints, making comparisons challenging [3, 8, 9]. Nonetheless, aggregate data consistently demonstrates improved functional capacity and HRQoL post- TAVI treatment for the majority of patients [10, 11].

Among the commonly employed non-disease-specific tools, the SF-36 questionnaire stands out for its widespread use and robust validation. This questionnaire offers insights across various disease spectra, including cardiovascular conditions, and updated Norwegian norm data are available [3, 12–16]. Of the SF-36 domains, physical functioning (PF) emerges as particularly relevant for AS patients, capturing limitations in everyday physical activities. Additionally, role limitations due to physical problems (RP) proves highly relevant in evaluating disability levels among patients with AS [17].

A notable challenge in utilizing the SF-36 questionnaire for AS pertains to the absence of established cutoff values for determining clinically significant symptomatic improvement at the individual level. While expert consensus panels have defined clinically important changes for heart disease, such levels are yet to be established for AS assessments [16]. Using this consensus report, relevant literature, Norwegian normative data, and input from local experts in PROM (Patient- Reported Outcome Measures) research, we defined clinically meaningful improvements as a minimum increase of 15 points in PF or RP scores. Our study thus aimed to determine the proportion of patients experiencing clinically significant improvements in HRQoL based on this specified cutoff value for the PF and RP domains. We also explored the associations between changes in these HRQoL domains and clinical parameters, aiming to identify shared baseline characteristics among patients reporting clinically significant improvements in HRQoL.

## Material and methodology

### Material

A total of 103 patients with severe, symptomatic AS and preserved left ventricular ejection fraction (LVEF), scheduled for transfemoral (TF) TAVI at Oslo University Hospital HF between April 2017 and February 2020, were included in a prospective observational study. The data from this study was provided from an unpublished study aiming at determining the predictive value of dobutamine stress echocardiography and cardiac magnetic resonance imaging on 12-month outcomes. All patients provided informed consent in accordance with the Declaration of Helsinki, and the study was approved by the Regional Ethical Committee of South-Eastern Norway (REK ref 2017/94/REK Southeast C). It is registered at www.clinicaltrials.gov under “Better Patient Selection to Transcatheter Aortic Valve Implantation” (NCT03107923). Data from 50 patients in this cohort were presented in a feasibility and safety study investigating the use of dobutamine stress echocardiography in high-gradient AS [18].

**Fig. 1:**
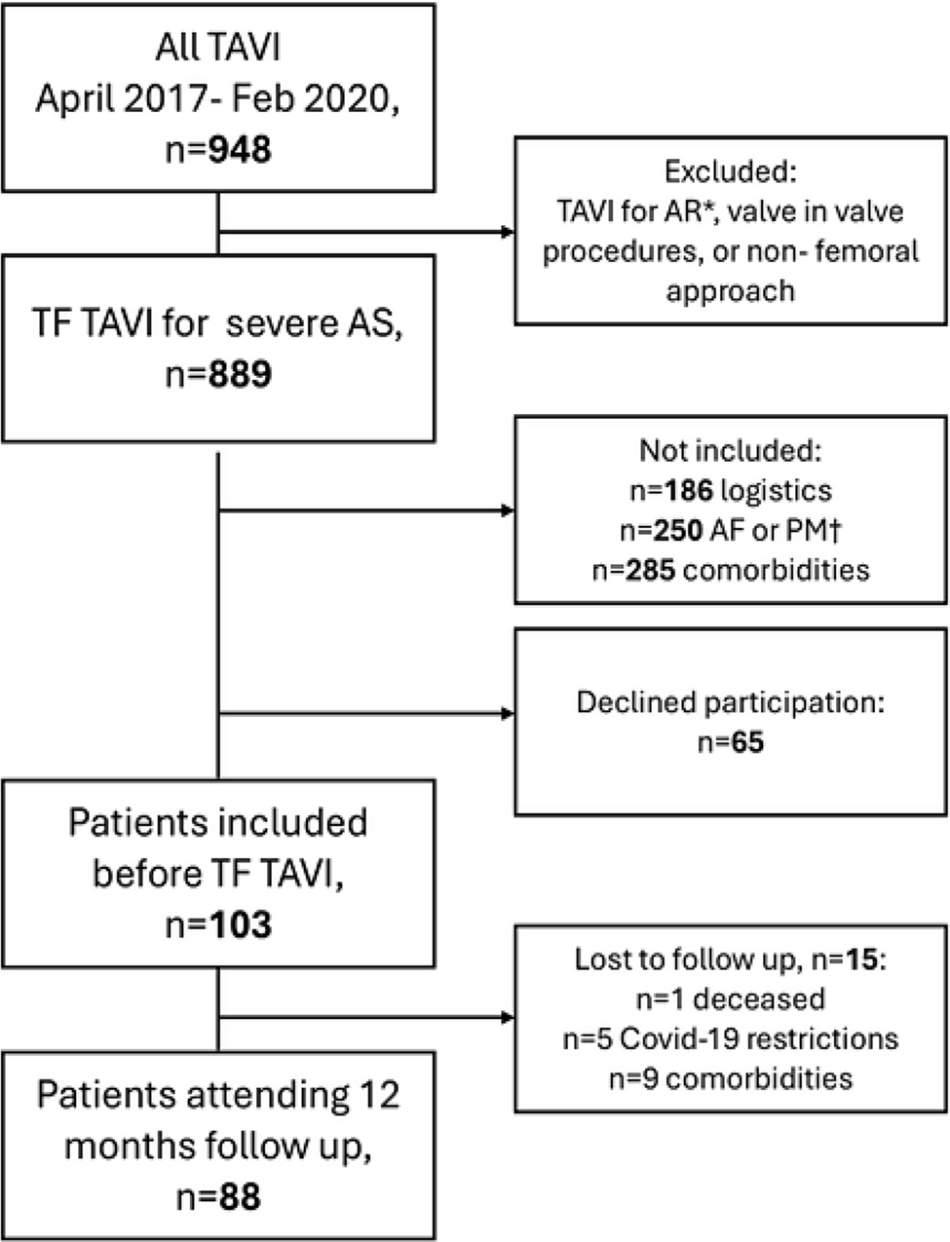
Flow chart for patient recruitment. TF TAVI Trans Femoral Transcatheter Aortic Valve Implantation. * AR aortic regurgitation. † AF atrial fibrillation, PM pacemaker

Inclusion criteria were severe, high-gradient AS approved for TF TAVI by the Heart team per ESC/EACTS guidelines [4, 5]. Exclusion criteria included clinical or hemodynamic instability, significant coronary artery stenosis, life expectancy under one year due to severe comorbidities, moderate or severe aortic/mitral regurgitations, chronic atrial fibrillation, pacemakers, inability to provide informed consent, and poor acoustic conditions preventing stress echocardiography interpretation (Fig. 1).

All patients were assessed on the day before scheduled TAVI, undergoing a comprehensive evaluation including clinical history review, physical examinations, laboratory tests, and transthoracic echocardiography. Subsequently, they underwent either dobutamine stress echocardiography, cardiac magnetic resonance imaging, or both [4, 19].

Echocardiography is the key tool for the diagnosis of AS, also providing prognostic information evaluating cardiac remodeling including left ventricular systolic function [4, 5, 19]. NT-proBNP (N-Terminal pro-B-type Natriuretic Peptide) correlates positively with severity of AS and symptom onset, and elevated levels tend to reflect advanced disease [20, 21].

Symptom severity and functional status were evaluated using New York Heart Association (NYHA) Functional Classification and 6-minute walking test (6MWT). The onset of symptoms in patients with severe AS signals a poor prognosis and is a Class I indication for aortic valve replacement based on international clinical practice guidelines [4, 5, 22]. The NYHA Functional Classification assesses the severity of dyspnea and functional limitations, strongly correlating with patients’ HRQoL [23]. The 6MWT provides an objective measure of the functional limitations in symptomatic AS, and the walked distance is independently associated with all-cause death or hospitalization for heart failure [24].

Operative risk was evaluated using EuroScore II and STS (Society of Thoracic Surgeons) Risk scores. Despite recommendations to use these scores, guidelines emphasize their significant limitations. As they were developed to estimate risk of mortality and morbidity after open cardiac surgery, neither score fully captures TAVI-specific factors like vascular access or procedural complexity, and they also lack measurements of frailty. The EuroScore II slightly overestimated 30-day mortality, while the STS score both underestimated 30-day mortality at lower predicted mortality scores and overestimated it at higher predicted mortality scores [4, 25].

Of the 103 patients, 88 patients received follow-up at 12 months where transthoracic echocardiography was performed, and NYHA Functional Classification and 6MWT results were reevaluated. As the primary focus was long-term outcomes after TAVI, no additional follow-up appointments were scheduled at Oslo University Hospital between 0 and 12 months.

### Health related quality of life

HRQoL was evaluated using the Norwegian version 2.0 of the SF-36 questionnaire, administered as a paper version to complete during consultations the day before and 12 months after TAVI. The results were entered into the Health Outcomes Scoring Software 5.0. The primary researcher managed all data collection.

The SF-36 questionnaire comprises 36 items across eight health domains: physical functioning (PF), role limitations due to physical problems (RP), bodily pain (BP), general health (GH), vitality (VT), social functioning (SF), role limitations due to emotional problems (RE), and mental health (MH). Each domain is assessed based on 2–10 questions, with scores ranging from 0 (worst health) to 100 (best health) [17].

The eight domains contribute to two main component scores: the physical component summary (PCS) and mental component summary (MCS) scores, standardized to a mean of 50 and a standard deviation (SD) of 10 in the general population [26]. Norm data are available for the general Norwegian population [15, 27, 28].

The most relevant SF-36 domain score in patients with severe AS is PF, as this domain measure limitations in behavioral performance of every day physical activities [17]. The domain role RP measuring the extent of disability in everyday activities due to physical problems, also was highly relevant in our patient cohort [17].

In general, there are two main approaches to finding minimal clinically significant differences (MCID) in a clinical setting. One is anchor-based methods, which involve literature and expert-based approaches, and the other is distribution-based methods, which involve statistical calculations, with a wide variety of methods available [29]. Traditionally, standards for assessing changes in patient-reported HRQoL have relied on a combination of statistical threshold values and qualitative evaluations [29]. Recently, more emphasis has been placed on clinical experience, and consequently clinically important changes in the SF-36 domain scores have been defined for heart disease by an expert panel [16]. The expert panel has presented a consensus report for heart disease where the majority of documentation is found for patients with coronary artery disease and heart failure, but no thresholds for minimal important change have been formally defined for assessing the subgroup of patients with AS.

Based on the consensus report for heart disease, supplemented by relevant literature from other disease groups and familiarity with Norwegian normative data on age-related changes in SF-36 domains, we established a cut-off of 15 points in PF or RP scores to define clinically meaningful improvement [15–17, 27, 28]. This threshold was further supported by consultations with local experts experienced in PROM research. Based on this cutoff value we determined the proportion of patients with clinically meaningful improvements in PF and RP from preoperative assessment to the 12-month follow-up.

### Statistical analysis

Statistical analyses were conducted using IBM SPSS Statistics for Windows, Version 28.0 (IBM Corp, Armonk, NY). Categorical data were presented as numbers (%) and compared using the Chi-square test. Continuous variables were assessed for normal distribution and presented as mean ± SD for normally distributed data or median (range) for skewed data. HRQoL data were presented as mean with 95% confidence interval (CI). Between-group comparison for normally distributed data was performed using independent samples t-test, while skewed data were analyzed with the Mann-Whitney U test. Statistical significance was set at *p* ≤ 0.05. Logistic regression and the Chi-square test were used to explore associations between changes in PF and RP and routine clinical parameters. There were no missing data for the PF and RP domains.

## Results

### Material

Of the initial 103 patients enrolled in our study, one patient died due to sepsis. Fourteen others were lost to follow-up (Fig. 1), though all were contacted by telephone. Baseline characteristics did not differ significantly between those lost to follow-up and those who attended follow-up visits. Travel restrictions imposed by the COVID-19 pandemic prevented five patients from attending; of these, four reported feeling healthier than before undergoing TAVI, while one, awaiting hip replacement surgery, experienced reduced walking ability unrelated to dyspnea. The remaining patients were unable to attend follow-ups due to physical comorbidities of varying severity, ranging from knee or back pain to one patient with terminal cancer. Additionally, one patient could not participate due to home caregiving responsibilities for a spouse with dementia.

The final cohort of 88 elderly individuals had a mean age of 80 ± 6 years, 49 (56%) being male, and with a low to intermediate estimated complication risk (Table 1). Notably, there was a high prevalence of hypertension, kidney failure, diabetes mellitus, and cardiovascular disease. All patients had severe, high-gradient AS, with preserved median LVEF of 65% (range 35–79%) (Table 2). All presented shortness of breath as their primary symptom, with half of the cohort classified as NYHA II and the remaining half as NYHA III.

**Table 1:** Comorbidities including preoperative risk scores for all patients included pre-TAVI (*n* = 103), and for those attending the 12 months follow up (*n* = 88)

	Included pre-TAVI, *n* = 103	Patients attending 12 month follow up, *n* = 88
Hypertension, numbers (%)	67 (65%)	56 (64%)
Cerebrovascular disease, numbers (%)	12 (12%)	12 (14%)
Myocardial infarction, numbers (%)	12 (12%)	12 (14%)
Coronary bypass surgery, numbers (%)	10 (10%)	9 (10%)
Peripheral atherosclerosis, numbers (%)	12 (12%)	12 (14%)
Diabetes mellitus type II, numbers (%)	13 (13%)	11 (13%)
Kidney failure (eGFR < 60 ml/min), numbers (%)	31 (30%)	25 (28%)
EuroScore II, %	2.3 (0.9–20.1)	2.3 (0.9–20.1)
STS Risk Score, %	2.0 (0.8–10.4)	2.0 (0.8–10.4)
Gender: female/male, numbers (%)	45 (44%)/58 (56%)	39 (44%)/ 49 (56%)
Body mass index, kg/m2	25.7 ± 3.8	
Age, years	80 ± 6	80 ± 6

STS Society of Thoracic Surgeons

**Table 2:** Measurements preoperatively for all included patients (*n* = 103), with separate values for those attending the 12 months follow up after TAVI (*n* = 88)

	Included pre-TAVI, *n* = 103	Preoperative values follow up patients, *n* = 88	12 months postoperative, *n* = 88	p*
6-minute walking test, meters	363 ± 111	370 ± 113	410 ± 112 (*n* = 85)	0.020
NT-proBNP, ng/L	486 (50-6045)	449 (50- 6045)	255 (50-7694)	< 0.001
NYHA- class I/II/III/IV	0/48/55/0	0/44/44/0	51/30/5/2	< 0.001
Ejection fraction ad mode Simpson, %	65 (35–79)	65 (35–79)	62 (25–79)	0.040
Aortic V max, m/s	4.7 ± 0.5	4.7 ± 0.5	2.4 ± 0.4	< 0.001
Aortic mean gradient, mmHg	58 ± 15	57 ± 14	13 ± 4	< 0.001
Indexed aortic valve area, cm^2^/m^2^	0.4 ± 0.1	0.4 ± 0.1	0.8 ± 0.2	< 0.001

*p-value from comparing preoperative and 12-month data for 88 patients

NT-proBNP N-Terminal pro-B-type Natriuretic Peptide, NYHA New York Heart Association

The median length of hospitalization was 6 days (range 2–19 days) for all 103 patients, and for the 88 attending the 12 months follow-up. The majority of the patients were discharged directly to their homes. The perioperative and long-term complication rates were low. During the TAVI procedure, three patients experienced life-threatening bleedings: one related to the vascular access site and two due to tamponade. However, all patients survived without long-term sequelae. One patient suffered a disabling periprocedural stroke, while another had a stroke during the follow-up period. Additionally, one patient required rehospitalization due to prosthetic valve endocarditis, and four patients needed permanent pacemakers.

Aortic valve function was found to be satisfactory in the majority of patients at the 12-month follow-up (Table 2). Patients were assessed to having a significant relief in their main symptom of dyspnea, with a notable improvement in NYHA class; 60% were classified as NYHA class I (*p* < 0.001). Moreover, they also demonstrated a significant improvement in the 6MWT, with an increase in distance from 370 ± 113 to 410 ± 112 m at the one-year follow-up (*p* < 0.020) (Table 2).

### Health related quality of life

At the time of inclusion, our patient cohort exhibited considerably lower scores across all domains compared to the general Norwegian reference population and the 70–79 years cohort, but similar to the small subgroup of 80 + years [15, 27, 28]. The PCS was thereof notably reduced (40.97 (39.02–42.93)), while the MCS was similar to the reference population average of 50 points (50.19 (48.07–52.30)) (Table 3).

Prior to TAVI, the most affected areas of HRQoL were RP, VT, and PF, with mean scores of 48.51 (42.44–54.58), 43.96 (38.93-49.00), and 54.20 (49.40-59.01), respectively (Table 3). Conversely, domains less impacted by physical disease and symptoms, such as MH, SF and RE exhibited higher scores.

**Fig. 2:**
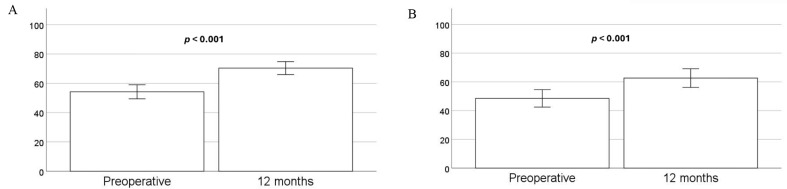
**A** Physical function scores preoperative and after 12 months, *n* = 88. **B** Physical role scores preoperative and after 12 months, *n* = 88. Data presented as mean with error bars for 95% CI

12 months post-surgery, patients reported statistically significant improvements in five SF-36 domains: PF, RP, GH, VT, and SF, along with the PCS (Table 3; Fig. 2). The most significant improvements were seen in the PF and RP categories, followed by a moderate improvement in the PCS. Despite these improvements, the PCS remained below the population average of 50 points, but better than the latest norm material of Norwegians over 80 years (average PCS of 37.02). PCS in our cohort after TAVI, aligned more closely with the 70–79 age group average of 42.47 points (Table 3) [27].

There were no significant differences observed between males and females in their reported HRQoL, both preoperatively and at the 12-month follow-up.

Utilizing the predefined cutoff of a 15-point increase as a clinically significant improvement in the PF category, the patient group was divided into two equally sized groups, with 44 patients experiencing a favorable outcome and 44 with an unfavorable outcome. Three patients reported a reduction of more than 15 points in PF.

Patients who experienced a more than 15-point increase in the PF category reported significantly lower PF scores before their valvular intervention (43.07 (37.37–48.78) vs. 65.34 (59.01–71.68), *p* < 0.001). This trend changed after 12 months, with those achieving a favorable outcome reporting significantly higher scores (75.11 (68.94–81.28) vs. 65.70 (59.43–71.97), *p* = 0.025) (Fig. 2). There was a significant correlation between the increase of 15 points and baseline PF scores (MCID PF no/yes = 2.9 − 0.05·baseline PF, OR 0.948 95% CI (0.924–0.972, *p* < 0.001).

In a univariate analysis, no significant correlations were found between improvement in the PF category and potential explanatory parameters from the routine baseline examination included age, preoperative risk scores, 6MWT results, NT-proBNP levels, and echocardiographic parameters assessing aortic valve and left ventricular function. Similarly, there were no significant differences in the prevalence of preoperative comorbidities or medications when using the Chi-square test. However, there was a significant correlation with the NYHA class: individuals who experienced an improved PF had more pronounced functional dyspnea beforehand (*p* = 0.033) (Table 4 in Supplementary).

**Fig. 3:**
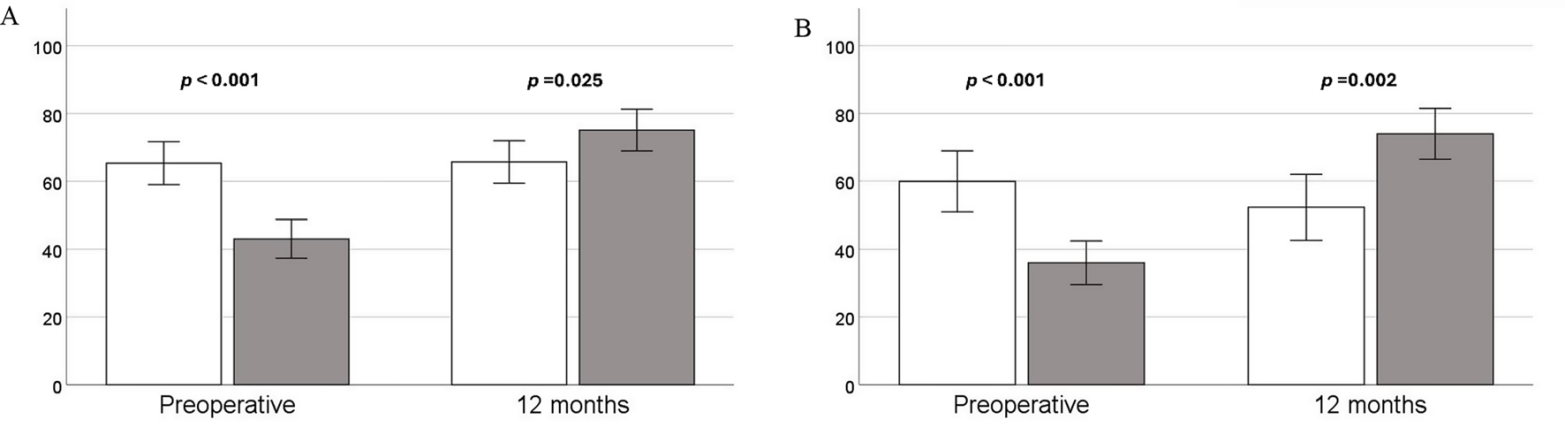
**A** Physical function scores preoperative and after 12 months, *n* = 88. **B** Physical role scores preoperative and after 12 months, *n* = 88. Data presented as mean with error bars for 95% CI. Patients divided by defined minimal clinically important change, with grey columns for patients increasing 15 points or more, and white without significant improvement

Similarly, using the 15-point MCID in the RP category, the patient cohort was divided, with 46 patients (52%) experiencing a favorable outcome. The remaining 42 patients had an unfavorable outcome, with 10 of them experiencing a decline of 15 points or more. Baseline RP scores were significantly lower in patients experiencing an increase of more than 15 points (Fig. 3), these preoperative RP scores were found to have significant associations with the presence of MCID in this domain (MCID RP no/yes = 1.5 − 0.03·baseline RP, OR 0.967 95% CI (0.958–0.984, *p* < 0.001). Again, the other baseline parameters mentioned earlier failed to do so, including NYHA class (*p* = 0.393) (Table 5 in Supplementary).

**Table 3:** SF-36 scores preoperatively and after 12 months for 88 patients

SF 36	Preoperative, *n* = 103	Preoperative, *n* = 88	12 months, *n* = 88	Change (95% CI)	p*
PF	52.62 (48.11–57.14)	54.20 (49.40- 59.01)	70.40 (65.98–74.83)	16.20 (12.03–20.37)	< 0.001
RP	45.93 (40.40-51.47)	48.51 (42.44–54.58)	62.64 (56.14–69.15)	14.13 (7.84–20.43)	0.001
BP	61.53 (56.05–67.01)	63.95 (58.14–69.76)	70.44 (65.04–75.83)	6.90 (1.49–12.30)	0.120
GH	61.63 (57.83–65.43)	62.06 (57.94–66.18)	67.65 (63.14–72.16)	5.59 (1.54–9.64)	0.028
VT	43.81 (39.33–48.29)	43.96 (38.93- 49.00)	54.47 (49.33–59.62)	10.51 (5.93–15.10)	0.004
SF	70.27 (65.30-75.24)	71.59 (66.19–76.99)	81.53 (76.77–86.60)	9.94 (4.85–15.04)	0.004
RE	69.17 (63.28–75.07)	71.69 (65.54–77.83)	80.21 (74.36–86.06)	8.52 (2.54–14.51)	0.053
MH	77.04 (73.72–80.36)	78.18 (74.59–81.78)	80.28 (75.95–84.62)	2.10 (-1.39- 5.60)	0.096
PCS	40.36 (38.55–42.17)	40.97 (39.02–42.93)	46.07 (44.23–47.90)	5.10 (3.19-7.00)	< 0.001
MCS	49.63 (47.72–51.55)	50.19 (48.07–52.30)	52.17 (49.87–54.46)	1.98 (0.13–3.82)	0.074

*p-value from comparing preoperative and 12-month data for 88 patients

BP Bodily pain, GH General health, MCS Mental component summary score, MH Mental health, PCS Physical component summary score, PF Physical functioning, RE Role limitations due to emotional problems, RP Role limitations due to physical problems, SF Social functioning, VT Vitality

## Discussion

Our study shows that treatment for severe symptomatic AS with transfemoral TAVI improves long-term self-reported HRQoL, results that are well in line with previous studies on this population [11]. The novelty of this study lies in our use of a cutoff value for determining clinical improvement in the PF and RP domains of the SF-36 questionnaire. Only half of the patients experienced such improvement 12 months after TAVI. Another important finding was that patients who had a more than 15-point increase in the PF and RP category reported significantly lower scores before TAVI. However, this trend shifted after 12 months, with those achieving a favorable outcome reporting significantly higher PF and RP scores (Fig. 3; Table 3). Moreover, it is of clinical interest that we did not find common characteristics for these patients in what constitutes standard preoperative assessment before TAVI. These findings indicate that HRQoL measures on physical health add valuable information on expectations to symptomatic benefit of treatment, and therefore, should be incorporated in the routine preoperative work up.

Two previous studies have used the SF-36 questionnaire to compare HRQoL in severe AS patients to the general population [2, 30]. Similar to these studies we found reduced preoperative physical and mental HRQoL in our TAVI cohort compared to the general Norwegian reference population aged 70–79 years and 80 + years [15, 27, 28]. One year after TAVI our cohort exhibited HRQoL values as good as, or better than, the general population aged 70–79 years. This was observed across all categories except vitality, which overall should be regarded as a favorable outcome in the patient cohort (Table 3).

For the individual patient with AS, there is no consensus of what should be regarded clinically significant improvement in the domain scores of SF-36 [7, 11, 16, 31]. This study is the first to use a threshold on clinically important difference or significant improvement in physical health symptoms based on this widely used questionnaire. Only half of the patients reported clinically significant improvement in physical symptoms based on our cutoff value of 15 points. Notably, few patients in our cohort had a significant clinical decline PF and RF with a decrease ≥ 15 points, and only one in 103 patients was dead at follow up. These findings allow for better and more graded evaluation of benefit of treatment of AS with TAVI, which is of importance for both clinicians and patients.

Left untreated, the prognosis for severe AS is poor with a high probability for a steep decline in physical health and risk of death even in a one-year perspective [22, 32]. Valve implantation is generally accepted as a low-risk, minimally invasive and lifesaving treatment, which disposes clinicians to offer TAVI and patients to accept the treatment. The ESC guidelines clearly state that the Heart Team is responsible for determining the optimal valve replacement treatment modality. While HRQoL is considered in this decision-making process, it is not the sole determining factor. The prognosis for symptomatic patients is poor without treatment, meaning treatment will likely proceed regardless of the HRQoL score. To what extent better information regarding physical HRQoL may affect patient selection to TAVI needs further investigations.

Our findings indicate that HRQoL measures on physical health add valuable information on expectations to symptomatic benefit of treatment. The patients who met the criteria for clinical improvement in PF reported significantly lower scores for the PF domain before valve implantation (Fig. 3). This also applied to the RP domain, as expected, since this category largely overlaps with PF in terms of questionnaire responses. Furthermore, patients who met the criteria for clinical improvement demonstrated significantly better HRQoL following TAVI compared to those who did not experience such 15 point improvement. Notably, individuals reporting the most impaired physical HRQoL preoperatively experienced the greatest symptomatic relief post-procedure. Hence, our findings support current guidelines offering TAVI to the most symptomatic patients as they appear to have the most to gain from it in terms of effective palliation of physical complaints [5].

To investigate whether these patient achieving significantly better HRQoL shared common preoperative characteristics, we examined parameters typically assessed in a standard preoperative evaluation, but found no significant correlations to age, comorbidities, gender, relevant blood tests, echocardiography, and walking distance in the 6MWT. NYHA functional class was before TAVI significantly higher in those who had MCID in PF, but the same could not be shown for those with improvement in RP. These results underscore that such patient reported HRQoL measures can add extra and relevant information to the routine preoperative work up.

Despite not being a disease-specific questionnaire, SF-36 has the advantage of covering general questions related to mental and emotional problems associated with poor health [3]. Preoperatively, the highest HRQoL scores were seen in parameters less affected by physical disease, the mental health domains RE and MH (Table 3). Awareness of the fatal nature of AS and the impact of related symptoms on mental well-being may been offset in patients accepted for TAVI due to promise of better survival and relief of symptoms [2]. The cohort had a score of MCS comparable with the Norwegian norm population of comparable age, likely reflecting a cohort with low prevalence of mental illnesses [27, 33]. These domains, and consequently the MCS, failed to show statistically significant improvement after 12 months, despite numerical increase. However, studies have demonstrated that TAVI surgery has been associated with improvements in psychosocial well-being as well, with patients commonly reporting reduced anxiety and depression levels post-procedure, along with an enhanced sense of overall life satisfaction when severe AS was alleviated [11, 31, 34, 35]. This trend was also observed in our patients, with a significant increase in GH, VT, and SF domains (Table 3).

Patients’ initial health status scores significantly influence changes over time. For instance, if a patient starts with a very high score on the questionnaire and experiences further improvement, this change may not exceed the threshold for clinical significance due to the questionnaire’s scoring limitations, though it may be very meaningful to the patient. Conversely, patients with very low initial scores might experience deterioration that does not register as clinically significant on the questionnaire, despite being very substantial from the patient’s perspective [16]. This may help explain that the PF and RP values for those with a significant increase above the 15-point threshold were initially lower before the operation. However, since the cohort as a whole reported values well below the average Norwegian population, the “ceiling” effect should not significantly influence the results.

The high number of non-responders in PF and RP domains could potentially be attributed to the presence of physical comorbidities, such as musculoskeletal disorders or lung diseases. This was not supported by our findings, as there were no significant differences in the 6MWT between responders and non-responder, both preoperatively and at follow-up. However, knee or back pain were the main reasons cited for patients not returning for follow-up assessments. This suggests that these physical ailments may hinder patients from achieving the HRQoL improvements despite potentially better cardiac function after undergoing TAVI.

Our study highlights the importance of defining a uniform cutoff value for clinically significant improvement in HRQoL using the SF-36 questionnaire. We chose an anchor-based method to determine the clinically significant change for the PF and RP domains in our paper, with emphasis on literature review including a consensus report on HRQoL for heart diseases, and discussions with experienced HRQoL researchers [16, 17]. Other results would have been somewhat different with the selection of alternative threshold values, but is beyond the scope of our article to go into detail of the multiple distribution-based methods to finding clinically significant changes in SF-36 domains. One of the most common statistical methods for estimating the threshold value for a clinically relevant change is using a change of ½ a standard deviation (SD) from the baseline value [29]. In our cohort of 88 patients, the baseline PF was 54.20 +/- 22.66, and RP was 48.51 +/- 28.66. This would result in cut-off values of 11.33 and 14.33, respectively, for the MCID after 12 months, and the values align well with our anchor-based threshold.

In our highly selected and relatively small AS cohort, the primary objective of the study was to determine the proportion of patients experiencing clinically significant, long-term improvements in HRQoL following TAVI. We choose univariate analysis for this specific purpose. Future studies involving larger populations will permit more comprehensive multivariate analyses to adjust for confounding factors and to provide deeper insights into which determinators that affect HRQoL improvements.

By examining preoperative HRQoL scores, patients can gain a clearer understanding of whether their HRQoL is categorized as poor or good before the procedure, helping to set realistic expectations for postoperative improvements. Notably, this emerging knowledge can be used to optimize their outcomes, including pre- and post-procedural rehabilitation [36, 37]. Prehabilitation before cardiac procedures and surgery is of gaining interest, utilizing the waiting period before elective cardiac interventions has proven beneficial for preventing further physiological decline, particularly in older patients [37]. Emerging research on prehabilitation prior to TAVI highlights similarly promising outcomes, including improved exercise tolerance and functional independence [36, 38]. The focus should be on protein rich nutrition, home based exercise, and worry reduction, to improve patient resilience in the preoperative period. However, it is crucial that prehabilitation efforts do not delay the TAVI procedure or extend the waiting time for this life-saving intervention.

HRQoL-related clinical improvements are important endpoints after TAVI, but it is important to note that not all patients awaiting TAVI will have poor HRQoL at the baseline evaluation. The focus should thus be on maintaining HRQoL in these patients, in addition to preventing further deterioration caused by symptoms or cardiac remodeling caused by AS.

Further studies should aim at establishing ground for a consensus on HRQoL related clinical improvements for patients with AS scheduled for TAVI. Our study is the first building block in this aspect. To further explore the utility of our proposed cutoff value, the next step could be to investigate the relations to patient frailty that is being frequently used in patient selection and risk assessment to TAVI. It would also be relevant to test SF-12 instead of SF-36, as it would be more accessible given its fewer questions and quicker administration.

### Limitations

A study strength was the complete data collection for all 88 patients before TAVI and at the 12-month follow-up, achieved by administering questionnaires during consultations and addressing missing responses immediately, eliminating non-responder bias. However, a limitation was the lack of questionnaires for patients who declined or were unable to attend follow-up, including those for whom travel to Oslo was too burdensome due to comorbidities.

Our elderly patient cohort makes it expected that some may not report a significant increase in physical aspects of HRQoL, and furthermore, one would expect a decline in physical health over time [18, 19]. A control group matched in age and gender would have been a strength for the study in this regard, providing even more insight into what cutoff for clinical improvement is most relevant.

A limitation of this study is the use of a non-disease-specific questionnaire to assess HRQoL in AS. While many different questionnaires are available for evaluation in this condition, incorporating disease-specific tools such as the TASQ or the KCCQ, both validated for AS and post-TAVI, would have strengthened the study [8, 9]. These tools could have better captured the relationship between changes in HRQoL and the relief of AS symptoms or the impact of comorbidities.

Our findings may not necessarily be reproducible in a cohort of patients with more advanced AS disease with reduced left ventricular ejection fraction, atrial fibrillation, pacemakers and higher preoperative risk scores, but align well with findings from the general TAVI population [39, 40]. The next step to further explore the relevance of the suggested cutoff threshold of 15 points would be to perform sensitivity analyses in a larger, real-life TAVI population [10, 41–43].

## Conclusion

Our study adds to the existing literature by highlighting the importance of defining a uniform cutoff value for clinically significant improvement in the SF-36 HRQoL questionnaire. Only half of the patients experienced clinically significant improvement 12 months after TAVI, and furthermore, patients that had poorest physical HRQoL preoperatively achieved the greatest relief in symptoms after the treatment. None of the routine preoperative tests were able to identify patients who would derive benefit in terms of HRQoL after TAVI, and thereof, SF-36 can provide supplementary information to the routine preoperative work up and follow up. This underscores the importance of considering individual patient characteristics when assessing patients with severe symptomatic AS.

## Electronic supplementary material

Below is the link to the electronic supplementary material.

Supplementary Material 1

## Data Availability

The datasets used and/or analyzed during the current study are available from the corresponding author on reasonable request.

## Abbreviations

6MWT: 6- Minute Walking Test
AS: Aortic Stenosis
BP: Bodily Pain
CI: Confidence Interval
GH: General Health
HRQoL: Health Related Quality of Life
KCCQ: Kansas City Cardiomyopathy Questionnaire
LVEF: Left Ventricular Ejection Fraction
MCID: Minimal Clinical Important Difference
MCS: Mental Component Summary Score
MH: Mental Health
NT-proBNP: N-Terminal pro-B-type Natriuretic Peptide
NYHA: New York Heart Association
PCS: Physical Component Summary Score
PF: Physical Function
PROM: Patient- Reported Outcome Measures
RE: Role limitations due to Emotional problems
RP: Role limitations due to Physical problems
SAVR: Surgical Aortic Valve Replacement
SD: Standard Deviation
SF: Social Function
SF- 36: Short-Form 36 Health Survey
STS: Society of Thoracic surgeons Risk Score
TASQ: Toronto Aortic Stenosis QoL Questionnaire
TF TAVI: Trans Femoral Transcatheter Aortic Valve Implantation
VT: Vitality
