# Supplementary material for: Health Related Quality of Life (HRQoL) after transcatheter aortic valve implantation in aortic stenosis patients: exploring a novel threshold for clinically significant improvement after 12 months
Source: J Patient Rep Outcomes. 2025 May 26;9:58. doi: 10.1186/s41687-025-00894-1 (PMC12106267; doi:10.1186/s41687-025-00894-1)
Supplement: Supplementary file 1 — Supplementary Material 1 [file 41687_2025_894_MOESM1_ESM.docx]

**Supplementary tables:**

Table 4: Correlation between increase above the 15-points threshold value for PF improvement, and baseline clinical and echocardiographic parameters for 88 patients.

| Baseline parameter | OR | 95% CI | *p* |
| --- | --- | --- | --- |
| PF | 0.948 | 0.924-0.972 | <0.001 |
| NT-proBNP, ng/L | 1.000 | 0.999-1.000 | 0.324 |
| 6-minute walking test, meter | 0.999 | 0.996-1.003 | 0.746 |
| STS Risk Score, % | 0.980 | 0.763-1.258 | 0.874 |
| EuroScore2, % | 1.002 | 0.896-1.120 | 0.975 |
| Ejection fraction ad mode Simpson, % | 1.009 | 0.956-1.065 | 0.751 |
| Aortic V max, m/s | 0.960 | 0.436-2.212 | 0.920 |
| Age, years | 0.973 | 0.903-1.049 | 0.482 |
| Gender, female/male |  |  | 0.283* |
| NYHA- class, I-IV |  |  | 0.033* |

**p- value from comparison of groups using Chi Square test.*

*CI Confidence interval, NT-proBNP N-terminal pro-B-type Natriuretic Peptide, NYHA New York Heart Association, OR Odds Ratio, PF Physical function, STS Society of Thoracic surgeons Risk Score.*

Table 5: Correlation between increase above the 15-points threshold value for RP improvement, and baseline clinical and echocardiographic parameters for 88 patients.

| **Baseline parameter** | **OR** | **95% CI** | ***p*** |
| --- | --- | --- | --- |
| RP | 0.967 | 0.950-0.984 | <0.001 |
| NT-proBNP, ng/L | 1.000 | 0.999-1.000 | 0.302 |
| 6-minute walking test, m | 1.000 | 0.996-1.004 | 0.936 |
| STS Risk Score, % | 0.919 | 0.711-1.189 | 0.522 |
| EuroScore 2, % | 0.932 | 0.821-1.058 | 0.275 |
| Ejection fraction ad mode Simpson, % | 1.038 | 0.981-1.099 | 0.195 |
| Aortic V max, m/s | 0.665 | 0.297-1.489 | 0.321 |
| Age, years | 0.972 | 0.902-1.049 | 0.467 |
| Gender, female/male |  |  | 0.868* |
| NYHA- class, I-IV |  |  | 0.393* |

**p- value from comparison of groups using Chi Square test*

*CI Confidence interval, NT-proBNP N-terminal pro-B-type Natriuretic Peptide, NYHA New York Heart Association, OR Odds Ratio, RP Role Physical, STS Society of Thoracic surgeons Risk Score.*
